# Diphtheria seroprotection among Indonesian children: Community Health Surveys Riskesdas 2007, 2013 and 2018

**DOI:** 10.1371/journal.pone.0343396

**Published:** 2026-02-27

**Authors:** Monica Dwi Hartanti, Sunarno Sunarno, Novaria Sari Dewi Panjaitan, Armedy Ronny Hasugian, Rita Marleta Dewi, Sarwo Handayani, Masri Sembiring Maha, Tubagus Ferdi Fadilah, Firda Fairuza, Nathalia Ningrum, Meiriani Sari, Dita Setiati, Nia Nurul Aziza, Arleen Devita, Donna Adriani, Jihan Samira, Christina Safira Whinie Lestari

**Affiliations:** 1 Department of Biology, Faculty of Medicine, Universitas Trisakti, West Jakarta, Jakarta, Indonesia; 2 Center for Biomedical Research, Research Organization for Health, National Research and Innovation Agency (BRIN), Bogor, West Java, Indonesia; 3 Research Center for Preclinical and Clinical Medicine, Research Organization for Health, National Research and Innovation Agency (BRIN), Bogor, West Java, Indonesia; 4 Department of Paediatrics, Faculty of Medicine, Universitas Trisakti, West Jakarta, Jakarta, Indonesia; 5 Department of Microbiology, Faculty of Medicine, Universitas Trisakti, West Jakarta, Jakarta, Indonesia; 6 Department of Physiology, Faculty of Medicine, Universitas Trisakti, West Jakarta, Jakarta, Indonesia; Kintampo Health Research Centre, GHANA

## Abstract

Diphtheria remains a public health concern in Indonesia despite long-standing inclusion of diphtheria-containing vaccines in the national immunization program. This study assessed temporal trends in diphtheria immunity among Indonesian children aged 1–14 years, identified vulnerable age groups, and examined factors associated with seroprotection. We analyzed diphtheria IgG antibody data measured by ELISA from the Indonesian Community Health Surveys (Riskesdas) conducted in 2007, 2013, and 2018. The analysis included 6,622 children (2007), 7,110 (2013), and 7,203 (2018). Bivariate and multivariate logistic regression analyses were performed to identify determinants of seroprotection. Across all surveys, diphtheria IgG levels were lowest at ages 1–6 years, increased at ages 7–10 years, and declined again from age 11 years onward. Overall seroprotection ranged from 71.1% to 83.6%, with a two- to threefold increase in long-term protection observed in 2018 compared with 2007 and 2013. In multivariate analyses, complete DTP immunization consistently remained the strongest independent predictor of seroprotection among children aged 1–4 years (p < 0.05). Among children aged 1–14 years, maternal education (Riskesdas 2007) and household economic status (Riskesdas 2018) were also associated with seroprotection. Following the introduction of the DTP4 booster, higher diphtheria IgG concentrations (GMC 0.48 IU/mL to 0.77 IU/mL) were observed among children aged 1–2 years old in 2018 compared with earlier surveys (GMC 0.18 IU/mL to 0.33 IU/mL). Diphtheria immunity among Indonesian children remains suboptimal, with the highest vulnerability at ages 5–6 years and evidence of waning immunity after ten years of age. Ensuring complete routine DTP immunization is critical, and booster strategies may be considered to sustain long-term population immunity.

## Introduction

Diphtheria is an easily transmitted infectious disease and can cause outbreaks with 5–10% case fatality rate (CFR), especially in young children [1]. The main cause of diphtheria is *Corynebacterium diphtheriae* (*C. diphteriae*), a Gram-positive rod-shaped bacterium that produces diphtheria toxin which has a toxic effect on target cells. This disease is characterized by inflammation at the site of infection, especially in the mucous membranes of the pharynx, larynx, tonsils, nose, and also on the skin. In more severe cases, diphtheria is characterized by difficulty swallowing, shortness of breath, stridor (a high-pitched noisy breathing sound), and swelling of the neck that looks like a cow’s neck (bullneck). Fatal cases usually occur due to obstruction/blockage of the airway, and systemic effects such as heart muscle damage, as well as abnormalities in the central nervous system and kidneys. Transmission occurs due to inhalation of saliva droplets from sufferers when the sufferer sneezes or coughs or through contaminated objects [2–4].

The incidence of diphtheria is spread throughout the world, especially in low-and middle-income countries (LMICs). WHO reported that in 2023, global diphtheria cases reached 24,782 cases, a significant increase compared to the previous year, which was 10,027 (2022) and 8,659 (2021). The most cases of diphtheria were found in Africa and Southeast Asia. In this case, Indonesia has always been among the five countries with the most diphtheria cases in the world from year to year [5,6]. Although diphtheria vaccine has been routinely given in the form of basic and additional vaccines, until now diphtheria cases are still being reported in Indonesia. The number of diphtheria cases reported in Indonesia in 2007, 2013 and 2018 (the data collection period for this study) are as follows, 183, 775 and 1,026 respectively [6]. Nationally, in 2020–2022, there were 964 diphtheria cases reported from 34 provinces in Indonesia [7]. Global data also shows that most diphtheria cases in various countries are related to immunization problems [8].

To date, early immunization against diphtheria in children has been very effective in drastically reducing the number of deaths and morbidity related to diphtheria. WHO recommends vaccination of at least six doses, namely three doses of basic vaccine and three doses of booster vaccine containing diphtheria and tetanus toxoids [9]. In its implementation, the diphtheria vaccination program depends on the policies of each country [8]. Since the early 1990s, Indonesia has implemented routine childhood immunization with three primary doses of diphtheria–tetanus–pertussis vaccine (DTP3) administered during infancy. In Indonesia, the diphtheria vaccination schedule determined by the Ministry of Health (Kemenkes RI) is given at ages 2, 3 and 4 months old for the first, second and third dose respectively, followed by boosters at ages 18 months and elementary school students in the first, second and fifth grades (Bulan Imunisasi Anak Sekolah, BIAS) which is in line with the recommendation of Indonesian Pediatric Society (IDAI). In addition, IDAI also recommends the booster doses scheduled for children aged 5–7 years and 10–18 years [10,11]. This difference arises from operational and logistical adaptations in the national immunization schedule, rather than from conflicting policy guidance. Consequently, our study’s references to school-based boosters should be understood as reflecting the implementation of the DT preparation under the BIAS program, while the IDAI recommendation pertains to the broader pediatric vaccination guidance.

In Indonesia, the vaccination program for diphtheria was executed based on the Expanded Program on Immunization (EPI), which recommends a primary vaccination series of three doses of DTwP-HB-Hib at 2, 3, and 4 months, followed by a booster dose at 18–24 months [11]. In addition to the national DTP vaccination schedule, Indonesia experienced a large diphtheria outbreak in 2017, which affected multiple provinces and prompted extensive outbreak response immunization (ORI) campaigns targeting children and adolescents. This outbreak prompted large-scale outbreak response immunization (ORI) campaigns, which delivered supplemental diphtheria-containing booster doses to children and adolescents, including cohorts beyond the age range typically covered by routine immunization services. These emergency vaccination activities occurred shortly before the Riskesdas 2018 survey and are likely to have influenced population-level immunity profiles observed in that survey year. Accumulating evidence of waning immunity following the primary DTP series, combined with persistent diphtheria transmission, raised concerns regarding the adequacy of long-term protection. In response, a fourth booster dose of DTP (DTP4) was gradually introduced into the national immunization schedule and more systematically implemented prior to the Riskesdas 2018 survey.

Globally, the coverage of three dosages of DTP vaccination in recent years has decreased from 86% in the 2015–2019 periods to 83% in 2020 and 81% in 2021. The Covid-19 pandemic that occurred in 2019–2021 has disrupted routine immunization services worldwide and has had an impact on decreasing immunization coverage [12]. A study conducted in 2023 during the pandemic in Jakarta, Indonesia, among school-aged children showed that only 40.9% of 154 children sampled received the primary DTP vaccination, first booster, and second booster. This finding indicated that complete diphtheria vaccination coverage remains low. Meanwhile, children who received the first and second booster doses had significantly higher anti-diphtheria antibody levels than those who did not receive the two booster doses. Therefore, booster vaccination in school-aged children is crucial for boosting immunity and minimizing the risk of diphtheria outbreaks [13]. The main objective of the 2030 immunization strategy is to expand immunization services to reach children who have not been immunized or whose immunization is incomplete, and to reduce the current inequality in immunization status [14]. While administrative coverage of routine childhood immunization is reported to be high, less is known about population-level immunity across age groups and how protection wanes over time in the post–scale-up period. In particular, evidence from nationally representative serological data remains limited.

The Ministry of Health, Republic of Indonesia (Kemenkes RI) delegated the National Institute of Health Research and Development to conduct a national basic health survey periodically every five years (well known as Riskesdas), starting in 2007, 2013 and 2018, and one of its objectives is to assess the health status of the community and the determinants that influence it. Serological testing for diphtheria was among the parameters evaluated, alongside those for pertussis, tetanus, hepatitis, and other vaccine-preventable diseases [15,16]. The Riskesdas data in 2007, 2013 and 2018 showed that more than half of the respondents in each survey period had complete DTwP vaccination. Accordingly, the three Riskesdas surveys analyzed in this study (2007, 2013, and 2018) represent distinct immunization and epidemiological contexts. Riskesdas 2007 reflects a period dominated by routine DTP3 vaccination, Riskesdas 2013 captures an intermediate phase prior to widespread DTP4 implementation, and Riskesdas 2018 reflects the combined effects of DTP4 introduction and recent ORI activities following the 2017 outbreak.

Most previous studies have relied on surveillance data or outbreak reports, which do not directly measure immunity at the population level. In contrast, this study leverages nationally representative serological data collected through multiple rounds of the Basic Health Research (Riskesdas) survey to characterize age-specific diphtheria antibody profiles over time. In this study, further analysis of diphtheria serology data aims to obtain trends in children’s immunity to diphtheria in Indonesia over a period of more than a decade, identify vulnerable groups and factors that influence protection for planning diphtheria outbreak prevention programs. To our knowledge, this is one of the few studies in the region to examine long-term trends in population immunity using repeated cross-sectional serosurveys.

## Materials and methods

### Study design

This study involves an in-depth analysis of the data obtained from the Basic Health Research (Riskesdas) survey conducted in all provinces of Indonesia, 2007, 2013 and 2018. A cross-sectional study design was employed to conduct Riskesdas which representation of population data on a national scale. In particular, the 2018 survey was conducted after the nationwide diphtheria outbreak in 2017 and subsequent ORI campaigns implemented across many provinces. As a result, serological findings from 2018 may reflect both routine immunization and outbreak-related vaccination activities. This study did not attempt to disentangle the individual effects of routine immunization, booster introduction, or ORI, but instead describes population-level immunity patterns resulting from their combined influence. All datasets were obtained at September 9^th^ 2022 from the dataset management laboratory of the Health Development Policy Agency, Ministry of Health, Indonesia [15,16]. Dataset was obtained after submission of a research proposal detailed with the required variables, including the serology data linked to the public health dataset. Proposal was reviewed by internal reviewers. After being accepted, the access to raw dataset would be granted based on the necessity. The principal investigator of this research signed the data transfer agreement for not sharing any data to any third-party. Additionally, all related publications should acknowledge the particular institution. Authors did not have access to the information that can be linked to participants.

### Samples

The sampling frame was developed using calculations from the BPS-Statistics Indonesia using multistage sampling across the stages as seen in the Riskesdas national reports for the years 2007, 2013 and 2018 [17–19]. The results of structured interviews and measurements were then subjected to additional analysis using selected household and individual samples. The chosen samples, that were older than a year old, underwent blood sampling and serological testing. Especially for diphtheria antibody examination, it was conducted on child respondents aged 1–14 years in Riskesdas 2007, 2013 and 2018. Respondents represented urban and rural areas except respondents in Riskesdas 2007 only represented urban areas. The number of child respondents examined for diphtheria antibodies was 6,622 (Riskesdas 2007), 7,110 (Riskesdas 2013) and 7,203 (Riskesdas 2018). Among these numbers, there were toddler respondents (aged 1–4 years) as many as 1,536 (Riskesdas 2007), 568 (Riskesdas 2013) and 1,052 (Riskesdas 2018). Toddler respondents were analyzed separately regarding their DTP immunization status which was only asked to respondents aged 1–4 years.

### Diphtheria antibody test

Antibody examination for diphtheria was performed on serum collected using the Enzyme-Linked Immunosorbent Assay (ELISA) method, according to the protocol in the insert package. The examination kit used was Indec Diagnostics, Germany. The examination was carried out in the laboratory of the National Institute of Health Research and Development, Ministry of Health of the Republic of Indonesia. Antibody titer for diphtheria is expressed in IU/mL units, with the following interpretations; seronegative/ partial protection (titer <0.1 IU/mL); full protection (titer 0.1–1 IU/mL), long protection (titer >1 IU/mL) [4].

### Vaccination history assessment

Individual vaccination history was available only for children aged 1–4 years in the Riskesdas surveys. Vaccination status was ascertained using documented vaccination cards when available; in the absence of a record card, caregiver recall was used, in accordance with standard Riskesdas procedures. Complete DTP immunization was defined as receipt of all age-appropriate DTP doses recommended under the national immunization schedule at the time of the survey. Children with missing or unverifiable vaccination history were excluded from analyses involving immunization status. The proportion of missing vaccination-history data is reported in the corresponding tables. Because individual-level vaccination data were not available or considered reliable for children older than four years old, analyses involving vaccination status were restricted to the 1–4 year age group. For older children (5–14 years), individual-level immunization data were not systematically documented, particularly for doses received under earlier routine schedules or through outbreak response immunization (ORI) campaigns; therefore, vaccination status for these age groups was not recorded.

### Operational definitions

The criteria for urban and rural residence were determined by the BPS-Statistics Indonesia when selecting the respondent sample. Multivariate models assessing determinants of diphtheria seroprotection included vaccination status only for the 1–4 year age group. For older age groups, analyses were limited to serological outcomes and demographic variables, without inclusion of individual vaccination history. Where relevant, the age-specific serological patterns in older children were compared with available programmatic immunization coverage data at the provincial or cohort level. These comparisons were treated as ecological in nature and interpreted cautiously.

Nutritional status was calculated based on the Body Mass Index (BMI)-for-age Z-score formulation with cut-points of: Malnutrition/ Severe wasted: ≤ −3, Undernutrition/ Wasted: ≤ 2.0, Normal: −2.0–2, Overweight: > 2.0, Obese: > 3.0 [20,21]. Children aged 1−4, BMI was calculated according to the WHO reference [21], while for children aged over 4 years, BMI was calculated using the CDC reference [20]. The results of this grouping are then grouped again into three large groups for analysis, namely (1) Undernutrition, which is a combination of undernutrition and severe malnutrition, where BMI z-score < −2.0, (2) Normal nutrition: where BMI z-score + 2.0 to −2.0 and (3) Overnutrition: is a combination of overweight and obesity, where BMI z-score > +2.0. Meanwhile, the family’s economic status is calculated based on expenditure quintiles and grouped into five groups, then grouped again into three categories, namely quintile 1–2 (poor), quintile 3−4 (middle) and quintile 5 (rich). Complete DTP immunization status in the analysis of Riskesdas 2007, 2013 and 2018 was defined as having received three doses of basic DTP immunization (DTP3) given at the age of 2-3-4 months. This immunization was given together with the hepatitis-B vaccine in the form of a DTP-HB combo vaccine (2011), which then replaced by the pentavalent DTP-HB-Hib vaccine in 2015. There are three degrees of maternal education: low for those who did not attend school, medium for those who attended elementary school through high school, and high for those who had a diploma-1 (D1) or were enrolled in college.

### Statistical analysis

The analysis was conducted on the samples of Riskesdas 2007, 2013 and 2018, namely children aged 1–14 years. The BPS-Statistics Indonesia determined the weight value of each data for survey analysis. Using univariate analysis, the proportion of participants such as sex, area of residence, age, nutritional status, family economic status, maternal education status, and toddler DTP immunization status, were identified. The age variable was divided into two categories (1–4 year old and 5–14 year old) based on the diphtheria immunization status data availability. Pearson’s-chi-squared bivariate analysis was used to assess the relationship between respondent characteristics and other variables related to diphtheria antibody titers. Multivariate analysis was used to assess the relationship of the most significant (p < 0.05) variables and conducted using binomial logistic regression to determine factors independently associated with the dependent variable. The analysis steps began with selecting variables included in the binomial multivariate analysis with p < 0.25 in the bivariate analysis. Then, the analysis was conducted by removing variables above p > 0.05 one by one. The results showed no interaction or confounding. The Hosmer–Lemeshow goodness-of-fit test and multicollinearity variance inflation factor (VIF) test demonstrated the suitability of the equation model. The final results are presented as an adjusted odds ratio (AOR) with a 95% confidence interval (CI). The AOR value illustrates the strength of the association between the independent and dependent variables after controlling for other variables in the model. In this study, diphtheria antibody titers were divided into two categories: protective (≥0.1 IU/mL) and negative/partial protective (<0.1 IU/mL). Stata software version 16 was used for statistical analysis.

### Data limitation

Data analysis was only conducted on variables available in Riskesdas 2007, 2013 and 2018 and data related to antibodies against diphtheria. Immunization status data was only available for respondents aged 1–4 years. Specifically, Riskesdas 2007 data was only available in urban areas. The total number of samples for variables analyzed using bivariate and multivariate methods differed according to the completeness of the available data. The DTP4 vaccination history and the information of diphtheria cases were unavailable.

### Ethical consideration

The request for raw data, de-identified data were obtained via email (datin.bkpk@kemkes.go.id) from the Data and Information Center, Health Policy Agency, Ministry of Health, Indonesia (Kemenkes RI), the official data holder. The ethical clearance for this analysis study was obtained from Research Ethic Committee, Faculty of Medicine, Universitas Trisakti, Jakarta with registry number No. 164/KER/FK/VIII/2022.

## Results

Characteristics of respondents aged 1–14 years who participated in the Riskesdas 2007, 2013 and 2018, and were examined for antibody titers against diphtheria can be seen in Table 1. In this study, the age range was divided into two; 1–4 and 5–14 years old based on the available immune status data in the Riskesdas database. The majority of respondents were in the 5–14 years age group for Riskesdas 2007, 2013 and 2018 (76.8%, 92.01% and 85.4% respectively). The proportion of respondents by sex was nearly equal. In Riskesdas 2007, all respondents lived in urban areas, while in Riskesdas 2013 there were more respondents living in rural areas, and vice versa in Riskesdas 2018. Associations between complete DTP immunization and diphtheria seroprotection are presented only for children aged 1–4 years, for whom individual vaccination history data were available. Most toddler respondents had complete basic immunization of DTP for Riskesdas 2007, 2013 and 2018 (67.1%, 74.6% and 78.3% respectively). Although there were still respondents with under nutrition status, most respondents had normal nutritional status for Riskesdas 2007, 2013 and 2018 (77.7%, 85.1% and 84.3% respectively). Based on family economic status, most respondents were at the poor to middle level (36.9% − 47.9%). The highest maternal education level was in the medium group for Riskesdas 2007, 2013 and 2018 (77.7%, 80.2% and 82.5% respectively).

**Table 1:** Distribution of the respondents aged 1-14 years old based on their characteristics.

		*Riskesdas 2007		Riskesdas 2013		Riskesdas 2018	
Characteristics	Value	*n* unweighting	%	*n* unweighting	%	*n* unweighting	%
Age		6,622		7,110		7,203	
	1-4 y.o		23.2		7.99		14.6
	5-14 y.o		76.8		92.01		85.4
Sex		6,622		7.110		7,203	
	Boy		50.6		48.4		51.7
	Girl		49.4		51.6		48.3
Areas		6,622		7,110		7,203	
	Urban		100.0		48.7		66.5
	Rural				51.3		33.5
DTP Immunization Status (1–4 y.o)		1,486		485		825	
	Complete		67.1		74.6		78.3
	Incomplete		32.9		25.4		21.7
BMI Nutrition Status		5,936		6,124		7,142	
	Undernutrition		14.0		9.2		7.3
	Normal nutrition		77.7		85.1		84.3
	Overnutrition		8.3		5.7		8.3
Family Economy Status		6,622		7,110		6,195	
	Poor		47.9		36.9		45.8
	Middle		38.2		47.7		36.9
	Rich		13.9		15.4		17.3
Maternal Education Level		5,822		6,324		6,474	
	Low		15.7		16.1		11.6
	Medium		77.7		80.2		82.5
	High		6.6		3.7		5.9

*Note* * Riskesdas 2007 was only conducted at urban areas.

**Fig 1:**
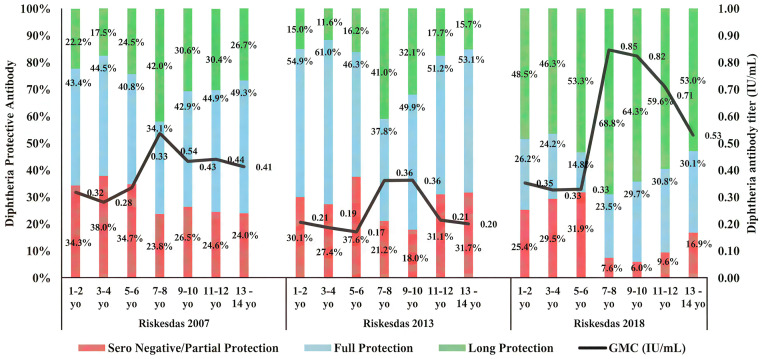
The trend of diphtheria titer in children aged 1-14 years, urban area on Riskesdas 2007, 2013 and 2018. Antibody concentrations (IU/mL) and corresponding protection categories (seronegative/partial, full or long protection) are shown across age groups for each survey year. Stacked bars present the distribution of seronegative/partial protection, full protection, and long-term protection. Antibody titer for diphtheria is expressed in IU/mL units. The seronegative/partial protection titer is defined as <0.1 IU/mL. Full protection titer is defined as 0.1-1 IU/mL. And long protection titer is defined as >1 IU/mL). The solid line represents the geometric mean concentration (GMC, IU/mL) for each age group.

Fig 1 generally shows a similar pattern in the three periods of Riskesdas. The proportion of respondents who had protective diphtheria antibody titers was still quite low at the age of 1–6 years, then increased at the age of 7–10 years, and decreased back simultaneously–often with the increasing age. The respondents living in urban areas in the Riskesdas 2007 showed a fairly high proportion of non-protective titers (<0.1 IU/mL) at the age of 1–6 years (34.7% – 38.0%). Meanwhile, long-term protective diphtheria antibodies (>1 IU/mL) increased with age, especially in the 7–8 group (42.0%), with the highest geometric mean concentration (GMC) at the age of 7–8 years (0.54 IU/mL), then gradually decreased (Fig 1).

The proportion of non-protective titers in Riskesdas 2013 was found to be lower than that in Riskesdas 2007 (18.0% – 37.6%). Meanwhile, long-term protective diphtheria antibody remained stable, but not as high as in Riskesdas 2007 in older age groups. The GMC in Riskesdas 2013 was lower than that in Riskesdas 2007, where the highest GMC was in the 7–10 year group (0.36 IU/mL) with a drastic decrease in the 11–14 year age group (Fig 1).

The proportion of non-protective titers in Riskesdas 2018 was found to be lower than that in both Riskesdas 2007 and 2013, especially in the 7–14 age groups. Meanwhile, the long-term protective diphtheria antibody level was found to be higher significantly, around two to three folds higher than those in Riskesdas 2007 and 2013, especially in the 7–12 age groups (59% − 69%). The highest GMC is at the age of 7–8 years (0.85 IU/mL) (Fig 1).

**Fig 2:**
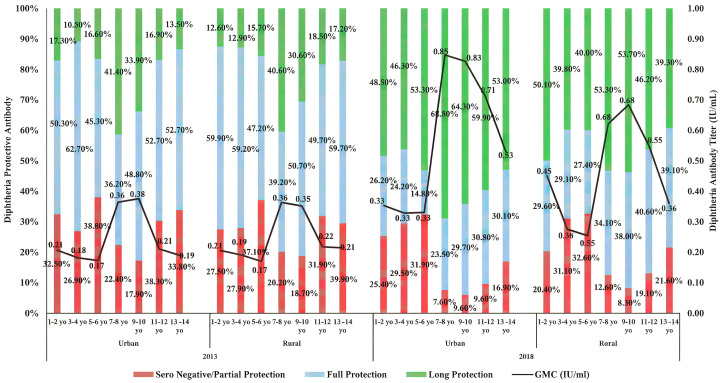
The trend of diphtheria titer in children aged 1-14 years, urban and rural area on Riskesdas 2013 and 2018. Stacked bars show the distribution of seronegative/partial protection, full protection, and long-term protection across age groups and survey years by urban/rural strata.

For respondents living in rural areas in Riskesdas 2013, most children have protective diphtheria antibody titers (0.1–1 IU/mL) (Fig 2). The proportion of children with non-protective titers (<0.1 IU/mL) is quite high at the age of 1–6 years (27%–37%) and increases again at the age of 11–14 years. Meanwhile, long-term protective diphtheria antibodies (>1 IU/mL) are relatively low in all age groups, with a high proportion in the age group of 7–10 years (30% − 40%). The GMC increases from the age of 7–8 years (0.36 IU/mL), then decreases slightly but remains quite stable until the age of 13–14 years (0.214 IU/mL). Meanwhile, the seroprotective diphtheria in urban and rural areas was found to be almost the same (Fig 2).

In Riskesdas 2018, the proportion of children with non-protective diphtheria antibody titers decreased, while the proportion of children with long-term protective diphtheria titers increased drastically, especially in the 7−10 year age group (about 53%). In addition, seroprotective diphtheria in urban areas was found to be higher than in rural areas. Protective diphtheria antibody titers increased linearly with age. The GMC titer is about two fold higher than that in Riskesdas 2013, especially in the 7−10 year age group (0.62–0.68 IU/mL). After the age of ten, the GMC began to decline but was still higher than that in Riskesdas 2013 (Fig 2).

Table 2 shows the results of the analysis to assess factors related to the status of diphtheria antibody protection in children aged 1–14 years in Indonesia. The percentage of protective diphtheria IgG antibodies is 71.1% (Riskesdas 2007), 72.6% (Riskesdas 2013), and 83.6% (Riskesdas 2018). These results indicate that around one-third of children in the 1–4 year age group were susceptible to diphtheria infection, with seronegative/partial protective diphtheria antibodies (diphtheria IgG < 0.1 IU/mL), both in the data of Riskesdas 2007 (36.4%), 2013 (28.4%) and 2018 (27.4%).

**Table 2:** Bivariate and multivariate analysis of diphtheriae immunity based on characteristic in children aged 1-14 years on Riskesdas 2007, 2013 and 2018^#^.

	*n*		Bivariate				Multivariate		
	*n* Weighting	*n* Unweighting	% Seronegative/ partial protective (<0.1 IU/mL)	% Protective (≥0.1 IU/mL)	POR	*p*	APOR	Lower- Upper	*p*
**A. Riskesdas 2007**									
**Age**									
1-4	364,351	1,230	36.4	63.6	ref				
5-14	1,236,593	4,372	26.7	73.3	2.74	0.000			
**Sex**									
Female	790,044	2,721	30.5	69.5	ref				
Male	810,899	2,881	27.4	72.6	2.74	0.000			
**Areas**									
Urban	1,600,943	5,602	28.9	71.1					
Rural									
**DTP Immunization Status (1–4 yo)**									
Complete	245,120	870	33.5	66.5	1.98	0.000	**1.46**	**1.1-2.00**	**0.020**
Incomplete	119,230	360	42.5	57.5	ref				
**Family Economy Status**									
Poor	772,704	2,708	30.1	69.9	ref				
Middle	612,974	2,129	27.3	72.7	2.67	0.000	1.02	0.70-1.47	0.932
Rich	215,265	765	29.5	70.5	2.39	0.000	0.64	0.39-1.05	0.078
**BMI Nutritional Status**									
Undernutrition	208,839	702	32.0	68	ref				
Normal nutrition	1,126,797	3,936	28.9	71.1	2.46	0.000			
Overnutrition	116,779	439	28.2	71.8	2.55	0.000			
**Maternal Education level**									
Low	216,292	681	32.3	67.7	ref				
Medium	1,095,486	3,851	28.6	71.4	2.50	0.000	1.38	0.78-2.46	0.266
High	89,265	387	29.3	70.8	2.42	0.000	**3.61**	**1.50-8.68**	**0.004**
**B. Riskesdas 2013**									
**Age**									
1-4	229,958	568	28.4	71.6	ref				
5-14	2,489,130	6,542	27.3	72.7	2.67	0.000			
**Sex**									
Female	1,317,226	3,446	27.4	72.6	ref				
Male	1,401,862	3,664	27.3	72.7	2.66	0.000			
**Areas**									
Urban	1,324,241	2,940	27.9	72.1	ref	0.000			
Rural	1,394,847	4,170	26.8	73.2	2.73				
**DTP Immunization Status (1–4 yo)**									
Complete	151,225	375	24.1	75.9	3.15	0.000	**2.02**	**1.09-3.74**	**0.027**
Incomplete	51,408	110	39.1	60.9	ref				
**Family Economy Status**									
Poor	1,003,564	2,980	30.6	69.4	ref				
Middle	1,296,652	3,061	25.7	74.3	2.89	0.000			
Rich	418,872	1,069	24.8	75.2	3.02	0.000			
**BMI Nutritional Status**									
Undernutrition	214,351	640	26.6	73.4	ref				
Normal nutrition	1,984,818	5,138	26.7	73.3	2.74	0.000			
Overnutrition	133,776	346	19.3	80.7	4.17	0.000			
**Maternal Education level**									
Low	394,893	1,153	26.9	73.1	ref				
Medium	1,966,464	4,916	27.6	72.4	2.27	0.000			
High	90,822	255	21.2	78.8	3.03	0.000			
**C. Riskesdas 2018**									
**Age**									
1-4	590,061	991	27.4	72.6	ref				
5-14	3,461,039	6,207	14.6	85.4	5.86	0.000			
**Sex**									
Female	1,958,156	3,386	16.2	83.8	ref				
Male	2,092,944	3,812	16.7	83.3	4.99	0.000			
**Areas**									
Urban	2,696,317	3,752	15.7	84.3	ref				
Rural	1,354,783	3,446	17.9	82.1	4.59	0.000			
**DTP Immunization Status (1–4 yo)**									
Complete	385,919	657	21.4	78.6	3.68	0.000	2.19	**1.33- 3.60**	**0.002**
Incomplete	107,222	167	35.3	64.7	ref				
**Family Economy Status**									
Poor	1,585,208	3,010	19.4	80.6	ref				
Middle	1,276,772	2,261	15.2	84.8	5.59	0.000	1.76	**1.06-2.93**	**0.028**
Rich	600,335	919	12.8	87.2	6.8	0.000	1.26	0.66-2.39	0.479
**BMI Nutritional Status**									
Undernutrition	295,107	543	16.1	83.9	ref				
Normal nutrition	3,389,695	6,012	16.6	83.4	5.03	0.000			
Overnutrition	334,438	582	15.6	84.4	5.41	0.000			
**Maternal Education level**									
Low	427,220	795	20.6	79.4	ref				
Medium	3,045,551	5,268	16.0	84	5.25	0.000			
High	216,953	407	13.4	86.6	6.46	0.000			

*Note:* ^#^Multiple Regression Logistic Analysis, POR: Prevalence Odds Ratio, APOR: Adjusted Prevalence Odds Ratio.

In Riskesdas 2007 (Table 2A) bivariate analysis results found that age, gender, DTP immunization status, family socioeconomic status, nutritional status, and maternal education level were significantly associated with protective diphtheria antibody status (p < 0.05). Children aged 5–14 years were 2.74 times more likely to have significant protective diphtheria antibody status than children aged 1–4 years (POR = 2.74; p = 0.000). Boys also had significant higher of protective diphtheria antibody than girls (POR = 2.64; p = 0.000). In addition, children who received complete DTP immunization showed significant higher chance of protection compared to children with incomplete immunization (POR = 1.98; p = 0.000).

Family economic status also showed a significant relationship, where children from middle-class (POR = 2.67; p = 0.000) and rich (POR = 2.39; p = 0.000) families had significant higher protective diphtheria antibodies than children from poor families. Likewise, normal nutritional status (POR = 2.46; p = 0.000) and over nutrition (POR = 2.55; p = 0.000) had approximately 2.5 times the chance of having significant protective diphtheria antibodies compared to children with under nutrition nutritional status. Maternal education was also a significant factor, where children with highly educated mothers had a 2.42 times greater chance of having significant protective diphtheria antibodies than those with low-educated mothers (p = 0.000).

However, after multivariate analysis, only two factors remained significant as independent predictors of protective diphtheria antibody status, namely complete DTP immunization status and maternal education status. Complete DTP immunization status (APOR = 1.46; 95% CI: 1.10–2.00; p = 0.020), showed that children who received complete immunization were 1.46 times more likely to have significant protective diphtheria antibody status compared to those who were incomplete. Furthermore, children from mothers with high education status (APOR = 3.61; 95% CI: 1.50–8.68; p = 0.004), showed a significant increase in the protective diphtheria antibody compared to mothers with low education. While the economic status of middle class (APOR = 1.02, p = 0.932) and rich class (APOR = 0.64, p = 0.078) do not seem to show significant results, yet in the statistical test, this variable cannot be removed from the multivariate analysis. These results indicate that the relationship between socioeconomic status and protective diphtheria antibody status can be explained by the existence of other variables, especially the completeness of DTP immunization, which has a direct and strong influence on the protective diphtheria antibody status.

In Riskesdas 2013 and 2018 (Table 2B and 2C), based on bivariate analysis, it was found that child age, sex, area, DTP immunization status, socioeconomic status, nutritional status, and maternal education level were factors that played a significant role (p < 0.001) on protective diphtheria antibody status. Children with complete DTP immunization status were 2.02 times (Riskesdas 2013, Adjusted POR = 2.02; 95% CI: 1.09–3.74; p = 0.027) and 2.19 times (Riskesdas 2018, Adjusted POR = 2.19; 95% CI: 1.33–3.60; p = 0.002) more likely to have significant protective diphtheria antibody status compared to children with incomplete immunization. Other factors such as age, gender, residence, socioeconomic status, nutritional status, and maternal education were no longer significant in the final model, indicating that their influence on diphtheria antibody seroprotective status was not independent, but rather through other pathways such as access to immunization. Only complete DTP immunization consistently remained a strong independent predictor across all three surveys.

## Discussion

Differences in sample sizes across the three Riskesdas periods were primarily attributable to the expansion of sampling areas within provinces. These variations were appropriately addressed by applying sampling weights in accordance with the national survey design. As a result, the analyses preserve population representativeness, allowing valid inferences regarding immunization coverage and serological protection among the Indonesian child population across survey years. Based on further data analysis Riskesdas 2007, 2013 and 2018 the proportion of non-protective diphtheria IgG titers (<0.1 IU/mL) is quite high at the age of 1–6 years, possibly because there were still around 25–30% of toddlers who had not received complete diphtheria immunization (Table 1, Figs 1 and 2). These results are similar to research conducted by Le et al on the population in Vietnam, namely around 31.7% of children aged 0–5 years are susceptible to diphtheria [22].

During the data collection of Riskesdas 2007, 2013 and 2018, national immunization program for diphtheria in Indonesia was given to infants aged 2, 3 and 4 months as basic immunization. Furthermore, booster or follow-up immunization was given to children aged 18 months and elementary school students in the first, second and fifth grades [13]. For infants and children aged 18 months, the diphtheria vaccine was given in the form of a DTP-HB-Hib, diphtheria vaccine prepared together with tetanus, pertussis, hepatitis B, and *Haemophilus influenzae* type-B (Hib) vaccines. Meanwhile, for elementary school students, the diphtheria vaccine was given in the form of a DT vaccine preparation for elementary school students in the first grade, and the Td vaccine with a lower diphtheria toxoid concentration to avoid increased reactogenicity in the fourth and fifth doses was given to elementary school students in the second and fifth grades [23–25]. Therefore, in line with the national immunization program of diphtheria booster vaccination for elementary school students, according to Riskedas data, the diphtheria IgG antibody titer had increased again and was highest at the age of 7–10 years (Figs 1 and 2). However, after reaching the age of ten years old, there was a decrease in the titer of diphtheria IgG antibody. Meanwhile, the most vulnerable age group (with non-protective diphtheria IgG titer) is 5–6 years old (Figs 1 and 2) in all three periods of Riskesdas. Previous study reported by Yusoff *et al* (2021) on the population in Malaysia with a sample size of 3,317 respondents also showed the same results, where children aged 5–6 years were most susceptible to diphtheria infection [26]. These results suggest that diphtheria vaccination boosters should be considered for both 5–6 years old of age and adolescents.

Diphtheria seroprotection in Riskesdas 2007, 2013 and 2018 ranged from 71.1%−83.6%, consisting of full protection and long protection. Long-term protection is defined as an antibody concentration ≥1 IU/mL [4]. Previous study showed that five years post-vaccination the antibodies started to wane slightly, but still remained higher than pre-vaccination [27]. The percentage of diphtheria long-term protective antibody in Riskesdas 2018 is found to be two to three fold higher than those in Riskesdas 2007 and 2013, in both urban and rural areas (2018 compared to 2013) (Figs 1 and 2). This is likely due to the diphtheria outbreak resulting in an increase in the immune response due to exposure to natural toxigenic *C. diphtheriae* infection and the additional diphtheria immunization program. During 2017, there was a diphtheria outbreak in 170 districts/cities from 30 provinces with 954 cases and 44 deaths, continuing until 2018. So that in December 2017–2018 through the outbreak response immunization (ORI) program, diphtheria vaccination was carried out targeting children aged 1–19 years. Diphtheria vaccine was given in the form of DPT-HB-Hib preparations for children aged 1–5 years, DT vaccine for children aged 5–7 years and Td vaccine for children aged over seven years. ORI aimed to increase community immunity so that it can break the chain of transmission. ORI diphtheria was implemented in three rounds, where the first round was implemented in 12 districts/cities in three provinces, namely DKI Jakarta, Banten and West Java, with an average coverage of 68.36% with a total target of 7.9 million. Furthermore, it was gradually implemented in 11 other provinces with the criteria of districts/cities that still have new case reports, which often report diphtheria outbreaks during 2017, which have diphtheria deaths, which have low routine immunization coverage <90% [24]. This result is also in accordance with the research conducted by Hughes et al, which showed that although DTP immunization coverage was higher in districts with low diphtheria incidence, immunity was significantly lower compared to districts with high diphtheria incidence. The higher levels of seroprotection observed in 2018 likely reflect the combined effects of routine immunization, booster dose implementation, and emergency vaccination responses, rather than survey year effects alone. Accordingly, DTP4 introduction and ORI activities are best regarded as plausible contributors to improved population-level immunity. Given the cross-sectional nature of the data, causal attribution to specific interventions is not possible, and observed patterns should be interpreted as descriptive of cumulative programmatic impact. High immunity in districts with high incidence is most likely due to natural immunity obtained through exposure to toxigenic *C. diphtheriae* infection and receiving a diphtheria vaccination booster [28].

Bivariate analysis of Riskesdas 2007, 2013 and 2018 data showed several factors that influence diphtheria seroprotection in children in Indonesia, such as age, gender, immunization status, family economic status, nutritional status and maternal education level (Table 2). However, multivariate analysis in the three Riskesdas periods showed that complete DTP immunization status in children aged 1–4 years was a factor that significantly influenced (Riskesdas 2007: p = 0.020, Riskesdas 2013: p = 0.027, Riskesdas 2018: p = 0.002) diphtheria seroprotection.

Factors influencing the completeness of immunization status in children aged under five years include maternal education level. Mothers with higher educational levels are often associated with a better understanding of child health, including the importance of immunization. In addition, older maternal age is also a contributing factor, as it is associated with a greater ability to make independent decisions. Furthermore, the presence of professional midwives during childbirth, who provided antenatal and perinatal services, including immunization, also played a significant role [29–32]. Accessibility of healthcare facilities, such as Puskesmas (primary healthcare) or Posyandu (integrated health posts), were also reported to play a crucial role in increasing immunization coverage [33,34]. Adequate vaccine availability at healthcare facilities, along with friendly and informative healthcare workers who foster maternal trust in immunization programs, were also found as important factors contributing to increased immunization coverage [35–38].

In children who have completed the DTP3 immunization series, the highest GMC of diphtheria IgG antibodies was observed in one year old, with seroprotective proportions of 77% (Riskesdas 2007) and 81.37% (Riskesdas 2013). These proportions declined with age, reaching 67.69% (Riskesdas 2007) and 60.88% (Riskesdas 2013) by four years of age (S1 Table). However, data from Riskesdas 2018 indicated a slightly different trend: while seroprotective proportion of diphtheria was high in one year old (82.8%), it peaked at two years of age (89.75%) before declining to 72.94% by four years of age (S1 Table). Riskesdas 2013 data showed a decrease of IgG diphtheria titer (GMC 0.33 IU/mL to 0.18 IU/mL – S1 Table). Meanwhile, Riskesdas 2018 data showed an increase of IgG diphtheria in children aged 1–2 years old (GMC 0.48 IU/mL to 0.77 IU/mL – S1 Table). The rise in GMC levels observed in 2018 should be interpreted in the context of overlapping immunization activities. Although the introduction of DTP4 represents a programmatic strengthening of the routine vaccination schedule, the extensive ORI campaigns conducted during the 2017–2018 national diphtheria outbreak may also have contributed to the enhanced antibody response. Similar to findings from previous outbreak response evaluations, supplemental booster doses delivered through ORI are known to elevate population-level immunity, particularly in older children and adolescents who may have waning protection [39,40]. Given that vaccination data did not allow us to differentiate between doses obtained through routine services versus ORI, the relative contribution of DTP4 alone cannot be fully disentangled. Therefore, the 2018 GMC increase is best interpreted as the cumulative effect of both routine schedule enhancement and intensified outbreak response measures. Nonetheless, immunity appears to decrease again after two years of age.

We acknowledge that observed patterns of declining diphtheria antibody levels with age are consistent with waning immunity post-vaccination, as documented in multiple seroepidemiological studies across different settings. Systematic reviews of DTP-containing vaccines have demonstrated measurable declines in post-vaccination immunity for diphtheria over time, highlighting the potential need for booster doses to maintain protection beyond early childhood [41]. Age-stratified serosurveys in other populations reveal similar decreases in protective antibody titers in older children and adults in the absence of recent boosting, suggesting that immunity gaps may persist despite high routine coverage [26]. While these cross-sectional findings support consideration of booster strategies, definitive conclusions regarding optimal booster timing and causal programmatic effects require longitudinal cohort studies, program evaluations, or formal transmission and immunity modeling that explicitly account for waning dynamics and vaccination history [40]. These findings suggest that completing the diphtheria immunization series significantly enhances children’s immunity against diphtheria, but this immunity wanes over time.

Immunization that is given completely according to the recommended dose can trigger an optimal adaptive immune response so that it can form long-term immunity. Although high maternal antibodies in newborns can cause low antibody titers after two doses of diphtheria immunization, after completing three doses of primary immunization, most infants have protective antibodies and as many as 94–100% of children have minimal seroprotection (titer >0.01 IU/mL) [42]. These results confirm that complete basic immunization coverage according to the immunization schedule is very important. Meanwhile, data from the three periods of Riskesdas indicated that the coverage of complete basic immunization remains below 80% (Table 1), thereby underscoring the need to intensify efforts to increase the coverage of both the third (DTP3) and fourth (DTP4) doses of the diphtheria vaccine. By maintaining national immunization coverage above 80%, herd immunity or immunity in the community is expected to be achieved so that the threat of outbreaks in the future can be reduced [43]. A meta-analysis of 266 articles on diphtheria and 55 articles on diphtheria outbreaks showed that three doses of diphtheria toxoids were effective in preventing symptomatic disease by 87% and reducing transmission by 60%. However, vaccination alone is certainly not enough and needs to be accompanied by active surveillance and appropriate antibiotic treatment [44].

The apparent discrepancy between the IDAI recommendation and the school-based immunization program warrants clarification. According to IDAI, the second DTP booster is recommended at approximately five years of age, before school entry, to sustain immunity against all three antigens [10]. However, under Indonesia’s national school-based immunization program (Bulan Imunisasi Anak Sekolah, BIAS), children in Grade 1 of elementary school typically receive a DT (diphtheria–tetanus) vaccine, which omits the pertussis component. This difference arises from operational and logistical adaptations in the national immunization schedule, rather than from conflicting policy guidance. Consequently, references of our study to school-based boosters should be understood as reflecting the implementation of the DT vaccine preparation under the BIAS program, while the IDAI recommendation pertains to the broader pediatric vaccination guidance.

National evidence showed that closing the gap in vaccination coverage had proven to be more effective and cost-effective than controlling the outbreak itself, as was the case with the diphtheria outbreaks in East and West Java in 2017 and 2023 respectively [39,45]. In Riskesdas 2007, in addition to immunization status, low maternal education level was also an influential factor (APOR = 3.61, p = 0.004) on diphtheria seroprotection compared to higher education. Maternal education plays an important role in improving health knowledge, attitudes, and practices, including compliance with the child immunization schedule. Mothers with higher education better understand the importance of immunization and its schedule. Education is also often associated with the ability to access valid health information and better use of health services. Many studies have shown that maternal education is significantly related to the completeness or incompleteness of children’s basic immunization, which has an impact on increasing immunization coverage and the resulting immunity [46,47].

In Riskesdas 2018, seroprotection was also significantly associated with household economic status, with individuals from poor households showing different odds of seroprotection compared with those from middle-income households (APOR = 1.76; p = 0.028). From a social determinants of health perspective, socioeconomic status influences health outcomes through access to key intermediary factors, including education, healthcare services, and adequate nutrition. However, in the context of immunological protection, immunization completeness appears to be a more proximal and dominant intermediary determinant. Household economic status is closely linked to complete versus incomplete immunization, which in turn affects overall immunization coverage and levels of seroprotection. This relationship is supported by studies from Afghanistan and India, which reported that children from wealthier households had a 36% higher likelihood of receiving complete immunization compared with children from the poorest households, even after adjustment for potential confounders [48,49].

This study is based on available secondary data, which might lack specificity about the brand, lot consistency and exact formulation type of the vaccine [50]. Additionally, there was little information available regarding the circumstances in which vaccinations were given, such as whether they took place just in public health centers or also in private medical facilities which limits this study scope. Our capacity to evaluate possible variations in vaccination quality, handling, and administration procedures is hampered by the lack of these specifics. Secondarily, the observed variations in seroprotection levels could have been caused by regional variations in the types of diphtheria-tetanus-pertussis (DTP) vaccinations used. For instance, a higher prevalence of acellular pertussis (DPaT) vaccine usage in private healthcare settings, as opposed to whole-cell pertussis (DPwT) vaccinations more frequently used in public immunization programs, may be the reason for lower seroprotection rates seen in certain urban area. These variations in vaccine formulation and cold chain are known to affect profiles of immunogenicity, which might complicate the comparisons [51,52].

The limitation of this study is that individual-level immunization history was available only for children aged 1–4 years. As a result, findings related to complete DTP immunization cannot be directly extrapolated to older age groups. Interpretations for these age groups are therefore based on observed age-specific serological patterns rather than confirmed individual vaccination histories. Observed serological patterns among older children likely reflect cumulative programmatic effects and waning immunity, and any comparisons with immunization coverage data at broader levels should be interpreted as ecological. Additionally, Riskesdas 2007 included only urban respondents, which may reduce comparability with later surveys and introduce urban–rural bias when interpreting changes over time. These limitations should be considered when generalizing the findings, and future studies incorporating detailed vaccine registry data, heterologous immunity between DTP and Covid-19 vaccines and mixed-methods assessments of immunization practices are warranted to better elucidate the determinants of diphtheria seroprotection.

## Conclusions

Using nationally representative Riskesdas data from 2007, 2013, and 2018, this study identifies persistent immunity gaps against diphtheria in Indonesia, with a high proportion of children aged 1–6 years lacking protective antibody levels and a marked decline in diphtheria IgG titers after 10 years of age. Although population-level seroprotection improved in 2018 following the introduction of the DTP4 booster and ORI activities, immunity remains suboptimal. Across all survey years, complete DTP immunization consistently emerged as the strongest independent determinant of diphtheria seroprotection, highlighting the central role of achieving and sustaining complete routine immunization coverage. These findings underscore the need to strengthen routine DTP delivery and to optimize booster dose strategies, including consideration of earlier and adolescent boosters, to mitigate waning immunity and prevent recurrent diphtheria outbreaks.

## Supporting information

S1 Table Diphtheria immunity status in children aged 1–4 years old with complete DTP vaccination status in the Riskesdas 2007, 2013 and 2018. (DOCX)
