# Supplementary material for: Diphtheria seroprotection among Indonesian children: Community Health Surveys Riskesdas 2007, 2013 and 2018
Source: PLoS One. 2026 Feb 27;21(2):e0343396. doi: 10.1371/journal.pone.0343396 (PMC12948082; doi:10.1371/journal.pone.0343396)
Supplement: S1 Table — (DOCX) [file pone.0343396.s001.docx]

S1 Table. Diphtheria Immunity Status in children aged 1-4 years old with complete DTP vaccination status in the Riskesdas 2007, 2013 and 2018.

| Time Riskesdas | Age (years old) | % Seronegative/ partial protective (<0.1 IU/mL) | % Protective (≥0.1 IU/mL) | Geometric Mean Concentration (IU/mL) |
| --- | --- | --- | --- | --- |
| 2007 | 1 | 23 | 77 | 0.88 |
|  | 2 | 20.99 | 79 | 0.69 |
|  | 3 | 26.49 | 73.51 | 0.78 |
|  | 4 | 32.32 | 67.69 | 0.63 |
|  |  |  |  |  |
| 2013 | 1 | 18.49 | 81.37 | 0.33 |
|  | 2 | 42.56 | 57.44 | 0.18 |
|  | 3 | 23.41 | 76.59 | 0.26 |
|  | 4 | 39.12 | 60.88 | 0.15 |
|  |  |  |  |  |
| 2018 | 1 | 17.2 | 82.8 | 0.48 |
|  | 2 | 10.25 | 89.75 | 0.77 |
|  | 3 | 21.28 | 78.72 | 0.45 |
|  | 4 | 27.06 | 72.94 | 0.39 |
